# Effects of Speed, Orthosis, and Load on Calf Muscle Electromyography Signal During Treadmill Walking

**DOI:** 10.3390/sports13020047

**Published:** 2025-02-08

**Authors:** Yasha Nahreini, Monika Herten, Jens-Peter Stahl, Christoph Schönle, Marcel Dudda, Thomas Jöllenbeck

**Affiliations:** 1 BBT Group, Department of Orthopedics and Trauma Surgery, Marsberg-Paderborn, 33098 Paderborn, Germany; y.nahreini@bbtgruppe.de; 2 Department of Trauma, Hand and Reconstructive Surgery, University Hospital Essen, University of Duisburg-Essen, 45147 Essen, Germany; marcel.dudda@uk-essen.de; 3 Department of Trauma, Hand and Reconstructive Surgery, Klinikum Dortmund Nord, 44137 Dortmund, Germany; jens-peter.stahl@klinikumdo.de; 4 Faculty of Health, University of Witten/Herdecke, 58455 Witten, Germany; 5 Department of Sports Medicine, Orthopaedic Rehabilitation Clinic, Klinik Lindenplatz GmbH, 59505 Bad Sassendorf, Germany; 6 Department of Orthopedic and Trauma Surgery, BG Klinikum Duisburg, University of Duisburg-Essen, 47249 Duisburg, Germany; 7 Institute for Biomechanics, Klinik Lindenplatz GmbH, 59505 Bad Sassendorf, Germany; thomas.joellenbeck@klinik-lindenplatz.de; 8 Department of Exercise and Health, Psychology and Human Movement, Paderborn University, 33098 Paderborn, Germany

**Keywords:** Achilles tendon rupture, orthoses, walking speed, plantar flexion, surface EMG, calf muscles activity, M. soleus, M. gastrocnemius

## Abstract

Background: Achilles tendon rupture rehabilitation protocols often emphasize two key factors, namely plantar flexion and load restriction during the early recovery stages. However, we hypothesize that variations in walking speed also play a significant role in affecting the load on the Achilles tendon. This study aims to explore the combined impact of plantar flexion angle and walking speed on the surface electromyography (EMG) activity of the calf muscles. Methods: Surface EMG measurements on 24 healthy volunteers assessed the activity of the calf muscles (gastrocnemius lateralis, gastrocnemius medialis, and soleus). Participants walked on a treadmill using two designs of ankle foot orthoses set at three different angles of the ankle joint (mainly 0°, 15°, or 30° plantar flexion), as well as barefoot and in sports shoes. The tests were performed at full loads of 1, 2 and 4 km/h or with additional measurements at 1 and 2 km/h with a partial load of 20 kg. The walking speed of 4 km/h in sports shoes was used as reference, corresponding to the maximum load on the calf muscles during walking. Results: Both orthoses demonstrated a significant reduction in EMG activity by more than half even at a 0° setting and 1 km/h compared to walking barefoot or in sports shoes. However, as walking speed increased to 2 km/h and especially to 4 km/h, EMG activity significantly increased, approaching the level of walking with sports shoes at 1 km/h. The results indicated that even minor changes in walking speed had a significant impact on muscle activity, underscoring the importance of this parameter. Conclusions: This study suggests that walking speed should be considered a crucial factor in rehabilitation protocols for Achilles tendon ruptures, alongside plantar flexion and load restrictions, to optimize recovery outcomes.

## 1. Introduction

The global incidence of tendon ruptures is estimated to range from 80 to 90 cases per 100,000 individuals, corresponding to approximately 6 to 7 million cases annually [1]. Among these, Achilles tendon rupture (ATR) is the most prevalent tendon rupture type in the human body, with sporting activity accounting for 68% of ATR [2]. A global increase in ATRs has been observed in recent years [3,4,5,6]. For instance, in Finland, the incidence of ATRs rose from 17.3 to 32.3 per 100,000 person-years between 1997 and 2019 [7]. The majority of ruptures (32%) occur in the age groups between 30 and 39 years, followed by those aged 40–49 years (25%), 50–59 years (15%), and 60–79 years (9%), while 19% of cases occurred in individuals under 30 years [6].

In recent decades, the incidence of Achilles tendon ruptures has significantly increased worldwide [3,4,5,6,7,8]. ATR incidences in Northern Europe increased in Finland from 17.3 to 32.3 per 100,000 person-years between 1997 and 2019 [9], with a similar tendency in Denmark from 27 in 1994 to 31 in 2013 [6].

The available evidence consistently demonstrates that ATRs predominantly affect males. A nationwide study in the US involving 6677 ATR cases over the period of 2006–2020 found that approximately 80% of ATR cases involved men, with a significantly higher incidence rate in males compared to females [8]. This male predominance is attributed to both biological and biomechanical factors, as men generally typically have larger calf muscles, which places greater strain on the tendon, in addition to differences in tendon properties, such as elasticity and strength [8].

The rupture of the Achilles tendon is more prevalent in sports that involve a “jumping and landing” phase, such as handball, volleyball, basketball, tennis, squash, and badminton. The effects of both the jump and landing phases, as well as the direction of movement, significantly influence Achilles tendon (AT) loading [9]. When the jumping and landing phases are more precisely distinguished, the tendon is subjected to greater stress during the jumping phase. The study by Gheidi and Kernozek (2019) demonstrated that the tendon experiences higher peak loads during the push-off (jumping) phase compared to the landing phase. This is attributed to the generation of substantial force by the gastrocnemius–soleus complex to propel the body upward, which directly increases the strain on the AT [9]. After surgical treatment, the overall return-to-play rate in elite athletes was 61% to 100%, with a greater reduction in performance in sports involving explosive plantar flexion like basketball [10].

An approach, which is used for recovery in the initial post-operative period after ATR, is to secure the ankle into a plantar-flexed position using footwear or orthosis. This pointed foot position after surgery ensures the adaptation of the two tendon ends, facilitating the bridging of the rupture site by connective tissue and thus promoting undisturbed healing [11]. This approach is critical because the suture can compensate forces only to a limited extent. If the foot is extended arbitrarily, strong tensile forces are exerted on the suture site, which could result in elongation of the rupture site or even rupture of the suture [12]. Most elongation tends to occur between 2 and 6 weeks post-surgery and avoiding dorsiflexion beyond neutral during this period is crucial to minimize this risk [13]. The second danger of overloading the tendon suture is a strong activity of the calf muscles.

Increasing the plantar flexion angle alters the biomechanics of the ankle joint, shifting the functional range of motion and affecting the activation patterns of lower limb muscles. A greater plantar flexion angle reduces the effective length of the gastrocnemius–soleus complex during the stance phase of walking. This biomechanical adjustment can influence muscle activation patterns, as the muscle–tendon unit operates in a shortened state, potentially reducing peak activation while still maintaining adequate force transmission for gait. This adjustment is particularly relevant during rehabilitation, as it aids in protecting the healing Achilles tendon by limiting excessive elongation and strain during walking [14].

Even at 30° plantar flexion, the strong calf muscle could pull the tendon stumps apart, for example, when walking or running. The peak loads for the Achilles tendon were calculated by 3.9 times bodyweight (BW) during walking and 7.7 times BW during running [15].

A variety of footwear and orthosis is available on the market with the objective of ensuring a pointed foot position. This is achieved by either raising the heel of the orthopedic shoe or by means of an external angle adjustment.

The rehabilitation phase is critically important for a successful recovery from Achilles tendon ruptures, yet there is a notable lack of standardized and evidence-based rehabilitation protocols. A 2017 study by Frankewycz revealed significant variability across 243 post-treatment protocols used nationwide in Germany, with recommendations for load guidelines, range of motion (ROM), physiotherapy, orthosis selection, and angle settings varying widely. In particular, the angle settings varied between 0° (neutral) and 30° of plantar flexion, with some protocols recommending a more pronounced plantar flexion to facilitate tendon healing, while others opted for a more neutral position to avoid excessive strain [16]. Increasing the ankle plantar flexion angle during walking alters the biomechanics of the ankle joint by reducing the effective length of the gastrocnemius–soleus complex. This adjustment can influence the muscle–tendon unit’s activation, operating in a shortened state to limit excessive elongation and strain on the healing tendon while still enabling sufficient force transmission for functional gait.

Despite the diversity of rehabilitation concepts, most post-treatment schemes for Achilles tendon ruptures focus primarily on two key parameters, namely plantar flexion and load restriction, during the initial stages of rehabilitation.

A consensus statement from the GAIT study group recommends initiating postoperative rehabilitation with non-weight bearing for approximately 2.3 weeks, maintaining the foot in plantar flexion during the first 4 weeks, and avoiding range-of-motion exercises beyond neutral during this period. Stretching and eccentric exercises should commence no earlier than 12 weeks post-surgery [17].

Biomechanics play a crucial role in gait recovery following Achilles tendon repair. Alterations in gait dynamics are observed both short-term and long-term after an Achilles tendon rupture. A study indicated that gait patterns are affected two to five years post-injury, highlighting the importance of addressing biomechanical factors during rehabilitation [18].

The impact of heel lift or heightened dorsiflexion restriction on surface electromyo-graphy (EMG) activity in the calf muscles has been examined in several studies [19,20]. Additionally, a comparative analysis of different orthoses has been conducted [21]. To date, however, there have been no studies that have systematically investigated and compared the effect of orthoses and load on calf muscle tension at different walking speeds as a primary variable.

The objective of this study is to examine the calf muscle activity, which is measured non-invasively through surface EMG in healthy individuals wearing different shoe orthoses in various angular positions of the ankle joint and at different gait speeds. We hypothesize that beyond load restrictions and plantar flexion, the additional factors of walking speed significantly influences the load on the Achilles tendon.

## 2. Materials and Methods

### 2.1. Subjects

A total of 24 individuals participated in the study, of which 14 (58.3%) were men and 10 (41.6%) were women. The average age was 39.1 years with a range of 20–70 years. Participants were recruited from the staff of the clinic as well as from sports clubs. All subjects met the inclusion criteria of being adult and physically healthy volunteers without anatomical or neuromuscular limitations of the lower limb. All participants provided their written informed consent to take part in this study and its publication. Their personal data were pseudonymized. The study was conducted according to the Declaration of Helsinki and achieved ethical clearance from the Ethics Commission of the Medical Council.

None of the study participants had any previous experience with relief shoes. The order of the two orthoses used was randomized so that each study participant completed their first treadmill trials with a different orthosis model.

During the examination, the subjects were secured with a safety belt that was hooked into a frame above the treadmill in order to prevent any disturbance to the gait. The test subjects were not subjected to any extreme stress during the examination, thus eliminating the possibility of any adverse effects. The participants were informed of the procedure and potential risks associated with the examination, including the possibility of falling on the treadmill, which was mitigated by the use of a safety belt.

### 2.2. Test Principle—Surface EMG and Instrument Setting

The activity of the calf muscles (Musculus gastrocnemius lateralis and Musculus medialis, and Musculus soleus) was measured using surface EMG [22]. Surface EMG electrodes do not directly measure tensile stress; however, they capture the electrical activity of the muscles, which can be correlated with the mechanical stress experienced by the tendon during movement or loading. Several biomechanical studies show that during activities like running or walking, the Achilles tendon undergoes significant mechanical loading, typically through elongation due to muscle contractions [23,24,25]. While surface EMG is less precise than directly using a strain gauge measured via sensors onto the Achilles tendon, surface electromyography is also non-invasive and allows for measurements of muscle activities across various walking conditions, including different speeds, ankle angles, and weight-bearing levels, in diverse subjects.

**Figure 1:**
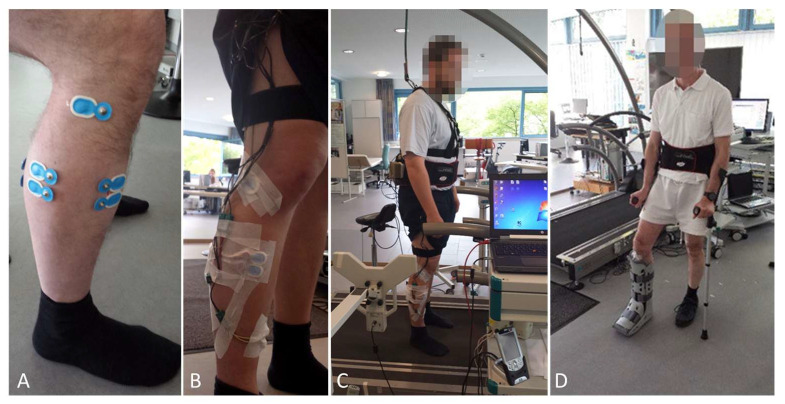
Experiment set-up. The electrodes are secured in position (**A**). The cables are attached to the leg (**B**). The participant is positioned on the treadmill wearing the electrodes and the belt (**C**). The participant exercises while walking with the orthosis and crutches (**D**) with partial weight bearing.

EMG electrodes were positioned at four precise locations on the lower leg to record muscle activity pertinent to the Achilles tendon, in accordance with the SENIAM guidelines with minor adaptations [26]. Leg hair and dead skin cells were removed to decrease resistance and improve electrode adhesion. Electrodes were placed consistently in line with the muscle fibers to obtain bipolar EMG signals between two electrodes on the muscle belly, capturing consistent muscle fiber activity. Differential amplification was used to reduce noise, and cables were securely attached to prevent detachment during movement. To minimize electrode positioning errors and ensure accuracy, electrodes were applied and remained in place throughout the experiment. This ensured consistent electrode placement, allowing valid comparisons between different walking speeds and therapeutic footwear. To reduce the effects of muscle fatigue which can affect EMG signals, the order of experimental conditions was randomized for each subject. Instrument settings are displayed in Figure 1.

### 2.3. Orthoses

Two different lower leg foot orthoses were used. The VACOped (OPED GmbH, Valley/Oberlaindern, Germany) is designed for the stabilization of injuries sustained to the foot [27]. The integrated vacuum cushion adapts to the anatomy of the foot and encloses swellings, thereby relieving pressure. A defined range of motion (ROM setting) permits freedom of movement for the injured joint as soon as the healing process allows. The range of motion is possible in 5° increments from 15° dorsiflexion to 30° plantar flexion. A lightweight, stable plastic shell provides support [27].

The AIRCAST^®^ Airselect™ Elite Walker (Enovis, Freiburg, Germany) is configured to provide immobilization in defined, adjustable positions and offers precise and secure embedding of the foot thanks to three individually inflatable air chambers [28]. An integrated air pump and air chamber system facilitate the reduction in pain and swelling. Two fixed half shells with individually adjustable Velcro straps provide high stability, while full foam padding in the shaft area creates a comfortable fit. The biomechanically optimized roll-off sole allows for ease of movement, while heel wedges permit a defined range of motion setting. Five anatomically shaped wedges of varying dimensions were provided for the establishment of the pointed foot position. Three wedges on top of each other correspond to an approximate angle of 22°; the two wedges below this correspond to approximately 16° [28].

### 2.4. Experiment Design

**Figure 2:**
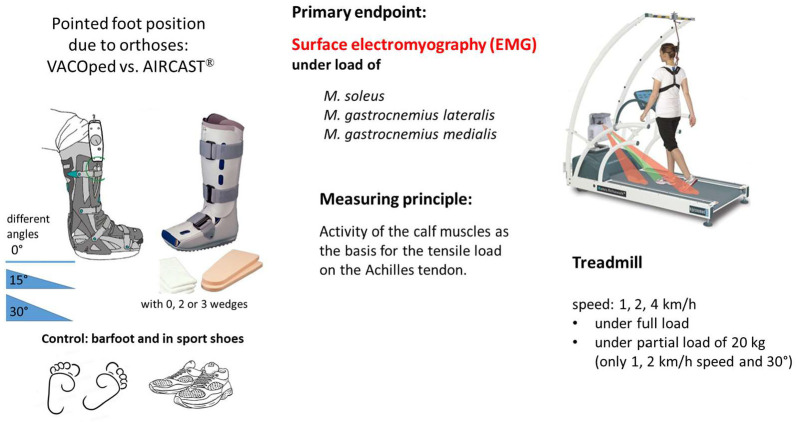
Experimental set-up.

**Figure 3:**
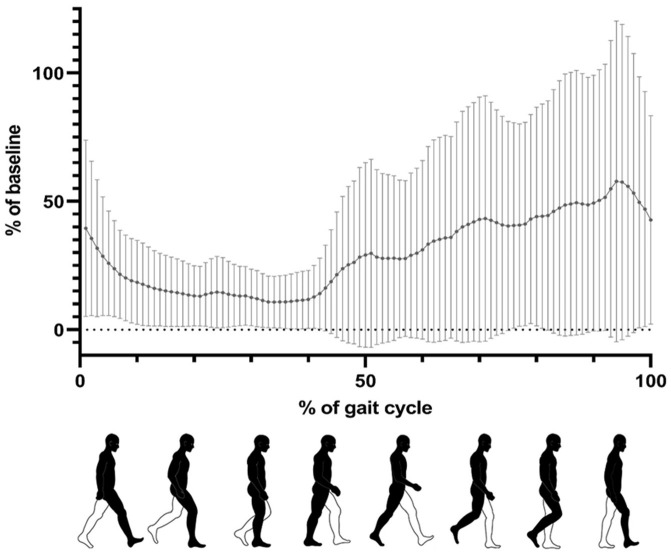
Example of a gait cycle during foot contact with bare feet at 0° of the Musculus gastrocnemius. One gait cycle is defined as 100%. The mean value (depicted in black) and the standard deviations (presented in gray) derived from the analysis of five to ten gait cycles per person and of n = 24 participants and were normalized to the baseline (sport shoes at 4 km/h = 100%). The lower part of the figure is adapted from [31,32].

An overview of the experimental set-up is shown in Figure 2. Subjects walked at full weight at different speeds of 1, 2, and 4 km/h using a treadmill with foot pressure measurements, primarily for precise speed control using two designs of ankle foot orthoses (VACOped or AIRCAST^®^) with three different angles (0°, 15°, and 30°) or wedge variations (0, 1, or 2 inserts), as well as barefoot and in sports shoes (Figure 3). Despite the angle settings of the AIRCAST^®^ Walker (0°, 16°, and 22°) not aligning precisely with the parameters of the VACOped orthosis (0°, 15°, and 30°), a comparative analysis was conducted below under the three angle definitions of “0°, 15°, and 30°”.

The detailed protocol is listed in Table 1. Subjects practiced walking at different speeds for 5 min before starting the measurements. Data collection began 30 s after speed adjustment. The primary goal of utilizing the treadmill was to ensure that participants reached and maintained the target walking speed. For partial weight bearing (20 kg), subjects were instructed in the use of forearm crutches and practiced walking on the treadmill for 3–5 min prior to measurement. A maximum walking speed of 4 km/h was chosen to simulate “full load” conditions, which are typically avoided during early recovery phases after Achilles tendon rupture. For each subject, the EMG curve measured at this speed with sport shoes was taken as the baseline for maximum muscle activity and tendon loading. All subsequent EMG data were analyzed relative to this baseline curve, providing insights into changes in muscle activity rather than absolute quantitative values.

### 2.5. Processing and Analysis of the Data

The data were collected on a treadmill (zebris FDM-T, zebris Medical GmbH, Isny, Germany) equipped with integrated measurement technology (zebris FDM, V.1.8, zebris Medical GmbH). EMG data were recorded according to SENIAM guidelines [26], with a sampling frequency of 1 kHz and a bandpass filter of 10–500 Hz, followed by rectification and the creation of envelopes using a low-pass filter at 5 Hz. After a familiarization phase of 30 s on the treadmill, the corresponding EMG curves of 5 to 10 step cycles were recorded to capture reliable and accurate EMG curves during steady-state walking rather than focusing on a fixed number of cycles. This approach allowed us to prioritize data quality over quantity, ensuring that only consistent and representative EMG signals were analyzed. The data were then averaged across all gait cycles of the measurement (each lasting 20 s). The EMG curves exhibited individual similarity for each subject, according to the step cycle and gait speed and were then summarized mathematically (Figure 3). The mean value, the standard deviation, minimum, and maximum were calculated from the summation envelope.

Surface electromyography inherently exhibits substantial inter- and intra-individual variability due to anatomical and physiological differences in muscle composition [29], as well as signal attenuation disparities caused by variations in subcutaneous adipose tissue thickness [30]. Accordingly, the gait pattern with sports shoes at 4 km/h was established as the 100% baseline for the sample-specific reference dataset. The EMG data were then correlated with the reference dataset of the gait pattern and calculated as a percentage of it. The EMG activity was calculated as the mean of several individual curves. These values were then averaged across all participants and depicted in the subsequent curves. To facilitate the comparison of different walking speeds, the gait cycle was represented not in units of time but as a percentage of the gait cycle during foot contact (Figure 3). The integral (area under the curve) of a complete curve of a gait cycle was calculated, which was averaged from all participants as a measure of muscle activity and compared. In the analysis, the amplitude and duration of activity of the lateral and medial gastrocnemius muscles were combined.

### 2.6. Sample Size Estimation and Statistical Analysis

Given the study design and the tests used, as well as the variability of the dynamic EMG data in intrapersonal comparisons with an amplitude-related variability of 4–10 %, a sample size of *n* = 16 was considered sufficient to demonstrate an effect of at least 15 % with sufficient power. Since the measurements were conducted under dynamic conditions on a treadmill, there is a theoretical risk of interference due to the wiring. To account for potential measurement errors and ensure the attainment of statistical significance with a cohort of healthy participants, the sample size was increased to 24.

For data sampling and analysis, Microsoft® Excel® (version 2016, Microsoft Corporation, Redmond, WA, USA) was used and SPSS (V 29, IBM, New York, NY, USA) for statistics. The descriptive statistics of the gait cycles comprised the mean, standard deviation, minimum, and maximum. The data representing the area under the curve of the gait cycle were tested for normal distribution using the Shapiro–Wilk test. The non-parametric data were described using the mean and the interquartile range. In order to investigate the influence of increasing walking speed (1, 2, and 4 km/h) or increasing angles (0°, 15°, and 30°), the paired non-parametric integral values were analyzed with the Friedmann test and Bonferroni post hoc test. For differences between the two orthoses (VACOped vs. AIRCAST^®^) and for the partial load (1 vs. 2 km/h), the Wilcoxon matched pair test was applied. The level of significance was set at *p* ≤ 0.05 (two-sided).

## 3. Results

### 3.1. Influence on Musculus soleus

**Figure 4:**
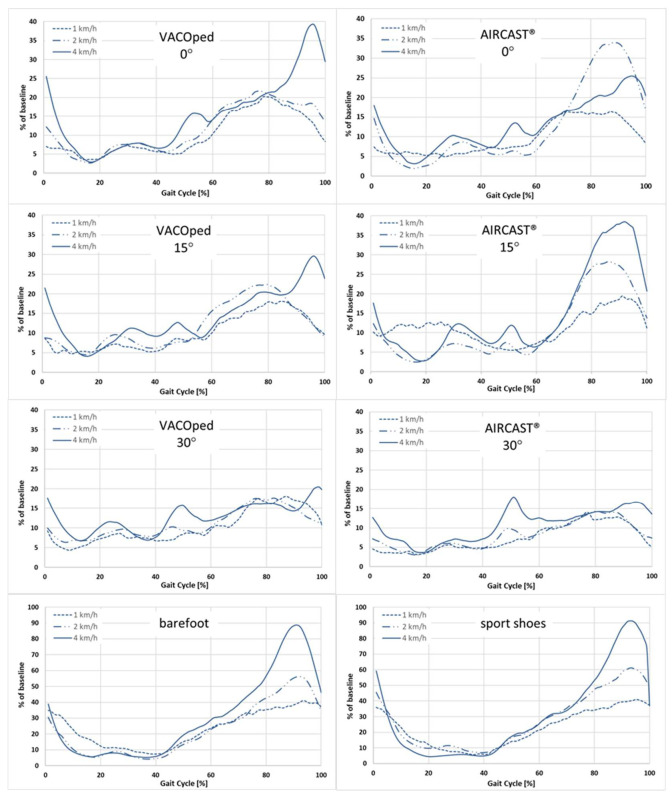
Impact of velocity (1, 2, and 4 km/h) on the EMG activity of the soleus muscle when wearing the VACOped or the AIRCAST^®^ orthosis and in barefoot and sports shoe conditions. The time is represented on the *x*-axis as a percentage of the gait cycle, with the surface EMG expressed as % of baseline (sport shoes at 4 km/h = 100%) at the *y*-axis. The mean values are derived from the analysis of five to ten gait cycles per person and of n = 24 participants.

The EMG activity of the soleus muscle was found to be reduced by more than half in both orthoses when compared to walking barefoot or wearing sports shoes (Figure 4). Regarding the scenario for the soleus muscle at an angle of 0° and irrespective of velocity, the mean values per gait cycle did not exceed 40% for both orthoses, whereas values of up to 90% were observed barefoot and in sports shoes. An increase in plantar flexion to 15° and 30° settings, irrespective of velocity, resulted in a reduction in soleus muscle activity in both orthoses.

For the AIRCAST^®^, the lowest values occurred at 15° and 30° and were about 20% regardless of the speed. With the VACOped, the values were observed to be below 30% at 15° and below 21% at 30°. At 30°, the muscle activity of the soleus muscle was reduced to approximately one-third of the level observed during free walking at the same speed.

Nevertheless, the EMG activity of the soleus muscle exhibited a notable increase with elevated walking speeds, irrespective of the type of footwear, including orthoses, sports shoes, and barefoot conditions. This was particularly evident at a velocity of 4 km/h, where the level of EMG activity approached that observed in the low-speed condition (1 km/h) during barefoot walking. An exception was the AIRCAST^®^ orthosis with an angle setting of 0°, in which the soleus muscle activity at a speed of 2 km/h was observed to be higher than that at 4 km/h (Figure 4).

**Figure 5:**
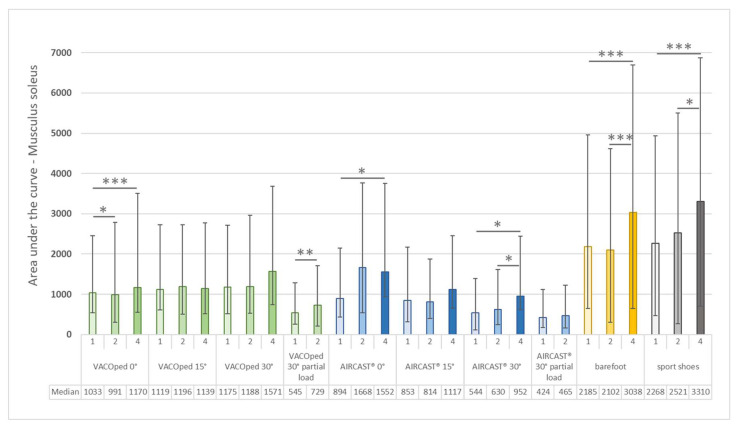
Musculus soleus—median and interquartile range of the integral of the area under the mean EMG curve of the gait cycles for both orthoses (VACOped and AIRCAST^®^) and of the controls (barefoot and sports shoes) at various angles (0°, 15°, and 30°) and at different walking speeds (1, 2, or 4 km/h) under full load or a partial load of 20 kg and 30° angle. The medians are presented in the final row. Significance levels: * *p* ≤ 0.05; ** *p* ≤ 0.01; *** *p* ≤ 0.001.

In relation to the integral of the gait cycle curve of the soleus muscle, a speed-dependent increase in muscle activity was observed for both orthoses and the control groups during walking barefoot or in sports shoes at full load (Figure 5). These differences were statistically significant regarding the angles of 0° for both orthoses and both control groups and also at 30° for the AIRCAST^®^ and for the VACOped at partial load. However, the differences were not significant for the VACOped at angles of 15° and 30° and for the AIRCAST at an angle of 15° and at partial load and 30° angle.

### 3.2. Influence on Musculus gastrocnemius

**Figure 6:**
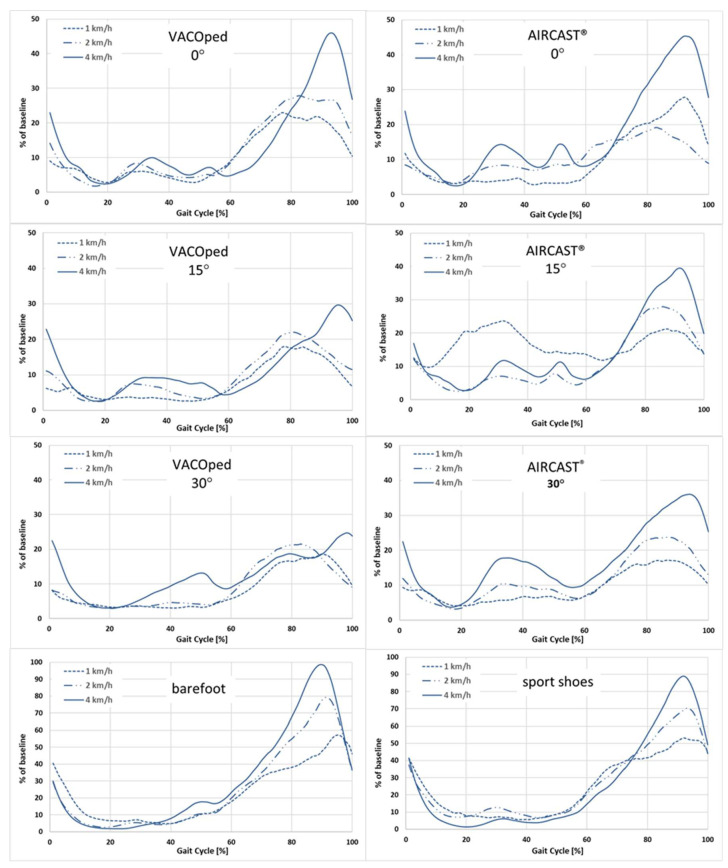
Impact of velocity (1, 2, and 4 km/h) on the EMG activity of the gastrocnemius muscle when wearing the AIRCAST^®^ or the VACOped orthosis, as well as in barefoot and sports shoe conditions. The time is represented on the *x*-axis as a percentage of the gait cycle, with the surface EMG expressed as % of baseline (sport shoes at 4 km/h = 100%) at the *y*-axis. The mean values are derived from the analysis of five to ten gait cycles per person and of n = 24 participants.

The results were comparable for the gastrocnemius muscle (Figure 6). The EMG activity was found to be also reduced by more than half in both orthoses when compared to walking barefoot or wearing sports shoes. Compared to the soleus muscle, the mean values per gait cycle were a little higher at up to 46%, while values of up to 90% were also observed in the absence of footwear and in sports shoes. Similarly to the soleus muscle, an increase in plantar flexion to 15° and 30° settings, irrespective of velocity, resulted in a reduction in muscle activity in both orthoses with the exception of the AIRCAST^®^ orthosis with an angle setting of 15°.

In the gastrocnemius muscle, EMG activity also increased significantly with increasing walking speed, regardless of the type of footwear, including orthoses, sports shoes, and barefoot walking. Again, this was particularly evident at 4 km/h, where the level of EMG activity approached that observed during barefoot walking at low speed (1 km/h). An exception was the AIRCAST^®^ orthosis with an angle setting of 15°, in which the gastrocnemius muscle activity at a speed of 1 km/h was observed to be partially higher than that at 4 km/h (Figure 6).

**Figure 7:**
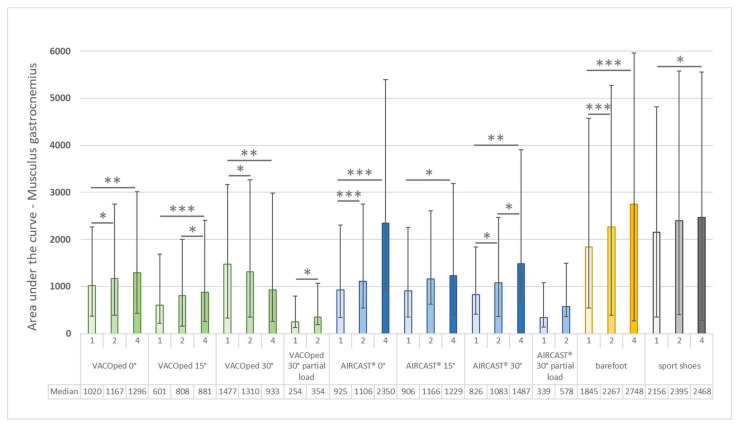
Musculus gastrocnemius—median and interquartile range of the integral of the area under the mean EMG curve of the gait cycles for both orthoses (VACOped and AIRCAST^®^) and of the controls (barefoot and sports shoes) at various angles (0°, 15°, and 30°) and at different walking speeds (1, 2, or 4 km/h) under full load or a partial load of 20 kg and 30° angle. The medians are displayed in the final row. Significance levels: * *p* ≤ 0.05; ** *p* ≤ 0.01; *** *p* ≤ 0.001.

With regard to the integral of the gait cycle curve of the gastrocnemius muscle, a speed-dependent discrepancy in muscle activity was discerned for both orthoses and the control groups when walking barefoot or in sports shoes at full load (Figure 7). These differences were statistically significant regarding all three angles (0°, 15°, and 30°) for both orthoses and both control groups and partial load for the AIRCAST^®^ but not for the VACOped. The muscle activities increased with increased walking speed, with the exception of the 30° angle for the VACOped, where the mean values of the integral declined significantly with increasing velocity.

### 3.3. Influence of Weight on Muscle Activity

**Figure 8:**
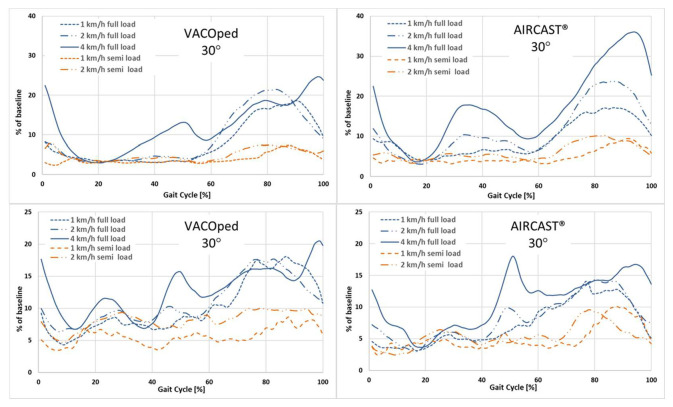
Comparison of the muscle activity of the gastrocnemius muscle (**upper row**) and the soleus muscle (**lower row**) when using the VACOped or the AIRCAST® orthosis under full or partial load in a 30° position at different speeds. The time is represented on the x-axis as a percentage of the gait cycle, with the surface EMG expressed as % of baseline (sports shoes at 4 km/h = 100%) at the y-axis. The mean value is derived from the analysis of five to ten gait cycles per person and of n = 24 participants.

The remarkable increase in EMG activity of the gastrocnemius muscles and the soleus muscle at elevated walking speeds was unmistakably illustrated through a comparative analysis of both orthoses during plantar flexion at a 30° angle with full and partial load (Figure 8). This phenomenon is solely contingent upon the velocity of the gait and is not influenced by the type of orthoses. The same holds true for walking barefoot or in sports shoes. The lowest EMG activity was recorded at a partial load of 20 kg at velocities of 1 and 2 km/h.

### 3.4. Differences in the Orthoses

In relation to the Musculus soleus activity, at an angle of 0° and a walking speed of 2 km/h, the VACOped shoe demonstrated significantly reduced M. soleus activity in comparison to the AIRCAST^®^ (*p* = 0.002) (Table 2). At lower speeds (1 km/h) and higher speeds (4 km/h), both orthoses exhibited comparable values. Additionally, at an increased angle of 15°, no discernible differences were observed between the two orthoses at any walking speed, a finding that was consistent at an angle of 30° and a speed of 4 km/h, as well as under the partial load condition at 1 km/h. However, a speed-dependent discrepancy in soleus muscle activity was identified at an angle of 30° and walking speeds of 1 and 2 km/h. In these conditions, the VACOped exhibited significantly higher soleus muscle activities (*p* = 0.001 and *p* = 0.020, respectively). Furthermore, at partial load and a speed of 2 km/h, the calf muscle activity of the VACOped was found to be significantly higher than that of the AIRCAST^®^ (*p* < 0.017).

With regard to the Musculus gastrocnemius, at an angle of 0°, significant differences were observed between the two orthoses only at 4 km/h, with the VACOped demonstrating higher muscle activity (*p* = 0.001). Further significant differences in muscle activities were present at an angle of 15°, with the AIRCAST^®^ showing significantly elevated values at both 1 and 4 km/h (*p* = 0.029 and *p* = 0.046, respectively). Conversely, at an augmented angle of 30° and walking speeds of 1 and 2 km/h and at partial load, the VACOped demonstrated significantly elevated soleus muscle activity (*p* = 0.017, *p* = 0.019, and 0.024, respectively). Furthermore, at a higher speed of 4 km/h, the muscle activity of the VACOped was found to be significantly higher than that of the AIRCAST^®^ (*p* < 0.037) (Table 2).

Overall, although some differences between the orthoses were statistically significant, they were marginal when compared to unassisted walking.

## 4. Discussion

This study examined muscle activity through surface EMG of the calf muscles in healthy individuals wearing different shoe orthoses in various angular positions of the ankle joint at different gait speeds and different weight bearings. As hypothesized, we could demonstrate that walking speed significantly influences calf muscle activity and, consequently, the stress on the Achilles tendon beyond load restrictions and plantar flexion.

The findings revealed that alterations in walking velocity exert a considerable influence on the calf muscle activity of both the gastrocnemius and the soleus, with or without the use of orthoses. An increase in walking speed from 1 km/h to 2 km/h and subsequently to 4 km/h was observed to result in a notable elevation in muscle activity across the majority of plantar flexion angle settings. Additionally, both ankle foot orthoses significantly reduce calf muscle activity by more than 50%, indicating a substantial reduction in calf muscle workload compared to the controls, which were walking barefoot or in sport shoes.

The unexpected findings of soleus and gastrocnemius muscle activity in the AIRCAST^®^ orthosis are considered a possible hypothesis based on biomechanical adaptations at lower walking speeds. At 0°, prolonged ground contact time and increased stability demands necessitate greater soleus activation, as the neutral ankle position reduces passive tendon recoil, requiring more muscle effort for stability and propulsion. Similarly, at 15° plantar flexion, the gastrocnemius, a biarticular muscle, plays a larger role in stabilizing the ankle and knee during the prolonged stance phase at very slow speeds. This increased activation likely compensates for reduced tendon energy efficiency. Furthermore, it is known that the gastrocnemius is not the primary muscle responsible for loading the Achilles tendon at slower speeds. These findings, though unexpected, align with the broader understanding of neuromuscular adaptations during gait and emphasize the complex interplay between walking speed, muscle activity, and orthotic settings.

A review of studies by Retting (2005) reported that patients under the age of 30 have a significantly higher re-rupture rate of approximately 16% compared to older age groups. This demonstrates that younger patients face a greater risk of re-rupture, even when following the same rehabilitation protocols as older individuals. This is paradoxical, as younger patients generally have better blood circulation and less tissue degeneration, conditions theoretically conducive to a more successful healing process. Nevertheless, the higher re-rupture rate in younger patients persists. Younger patients, due to their better overall physical condition, tend to regain mobility more rapidly and consequently reach higher walking speeds earlier in their recovery. This increased speed may place additional strain on the Achilles tendon, contributing to the higher re-rupture rates observed in this demographic [33].

Furthermore, partial weight bearing with either orthotic shoe led to a significant reduction in calf muscle activity, albeit by less than 50% compared to full weight.

Our results are in line with a study by Miyoshi et al., who measured the surface EMG activities of the soleus and gastrocnemius muscles and ground reaction forces in a reduced gravity environment in deep water [34]. The authors reported that with faster walking speeds, medial gastrocnemius muscle activity increased more than the soleus muscle activity. In the present study, the mean muscle activity values per gait cycle were also higher for the gastrocnemius muscle than for the soleus muscle.

A sono-tracking study of the Achilles tendon (AT) by Franz et al. (2015) performed ultrasound elastography to investigate in vivo deformations of the human free AT during treadmill walking. The authors could show that the differences between superficial and deep AT deformations enlarged with higher walking speeds (from 2.7 to 4.5 km/h). In particular, they found that faster walking induced a disproportionate increase in superficial compared to deep AT strain [35].

A recent review provides a summary of the current literature on the forces and strain acting on the Achilles tendon during dynamic exercise [36]. With regard to the techniques that have been employed in vivo to act on the human tendon in dynamic exercises used during rehabilitation, these have predominantly comprised heel raising [37,38,39,40,41,42] as well as squats [43,44].

To date, no studies have systematically investigated and compared varying walking speeds with orthoses as a primary variable. To the best of our knowledge, our study is the first to systematically investigate and compare varying walking speeds with orthoses as a primary variable on the calf muscle surface EMG.

The results of our study indicate that ankle foot orthoses significantly reduce calf muscle activity by more than 50% compared to sports shoes. This finding aligns with the research of Akizuki et al. (2001), who demonstrated that calf muscle EMG activity during ankle immobilization in a cam-walker and the insertion of a 1-inch heel lift resulted in a reduction in calf muscle activity to 57% of normal walking levels [19]. In their study, no variable speeds were tested.

Furthermore, Kadel et al. (2004) yielded comparable results in terms of reducing EMG activity in the calf muscles through the incorporation of additional wedges in two different shoe orthoses [20]. In addition, in this study, no variable speeds were examined, as the subjects walked at their own self-selected pace.

The findings of our study are closely aligned to a recent study by Fröberg et al. (2020), who investigated the impact of three different designs of ankle foot orthoses on non-uniform displacement patterns within the Achilles tendon, muscle activity in the lower leg, and plantar pressure distribution in healthy subjects [21]. Their measurement methods were EMG of the calf muscles, dual sonography, and foot pressure sensors at a walking of 2 km/h, which was normalized to mean peak EMG during unbraced running at 10 km/h. Additionally, this study examined the influence of allowing for varying degrees of dorsiflexion during walking on these variables. The authors found that differences in ankle foot orthosis (AFO) design did not significantly affect non-uniform Achilles tendon displacement or calf muscle activity. However, increasing dorsiflexion restriction within rigid and adjustable AFOs significantly reduced non-uniform displacement and soleus muscle activity. The study emphasizes that dorsiflexion allowance impacts biomechanics more than AFO design differences [21]. Despite the similarities in measurement methods between Fröberg et al.’s study and ours, their study examined only one speed at 2 km/h with orthoses, while our study specifically compared walking speeds of 1, 2, and 4 km/h.

In a scoping review about modeling and the in vivo evaluation of tendon forces and strain in dynamic rehabilitation exercises, Kharazi et al.’s (2023) study shares similarities with our research in its use of EMG and tendon force–elongation measurements across multiple walking speeds [25]. However, the key difference lies in their focus on high walking speeds (2.5, 5, and 7.2 km/h) and the distinct roles of the soleus and gastrocnemii muscles in enhancing ankle mechanical work. In contrast, our study used ankle foot orthoses and emphasized smaller incremental speed changes, the interaction between ankle angle and weight bearing, and their role in rehabilitation without addressing biarticular mechanisms.

Limitations of the study: The considerable standard deviation observed in this study suggests that participants did not consistently maintain a uniform gait, even after the exclusion of some extreme outliers from the calculations. This variability was particularly pronounced when participants walked with partial weight bearing using the orthoses, with the standard deviation exhibiting a marked increase relative to the mean values. The maintenance of partial weight bearing with two crutches proved to be a challenging task, resulting in fluctuations in ground reaction forces that are likely to have influenced muscle activity levels. A further limitation is that the sample is very heterogeneous in terms of age and ranges from 20 to 70 years old. This non-homogeneous group was intentionally selected to ensure that the findings of the study could be generalized to a broader clinical population. Achilles tendon ruptures occur across a wide spectrum of individuals, and a heterogeneous sample allows for the evaluation of rehabilitation strategies that are applicable to diverse patient groups in real-world settings.

Furthermore, the act of walking on a treadmill, which is an unfamiliar activity even for healthy participants, when combined with the additional challenge of using orthoses, may have contributed to these fluctuations. It can be reasonably inferred that not only the study participants but also patients lacking familiarity with walking in orthoses may initially experience difficulties in coordinating their movements. For patients undergoing surgery, it may be advisable to utilize walking aids for the initial two-day period following the procedure. This will facilitate the safe training of gait, including the prevention of thrombosis.

A methodological limitation of this study was the inability to monitor EMG curves in real time alongside treadmill data, including variables such as speed and ground reaction forces. Although the EMG data were recorded in real time, they could only be subjected to retrospective analysis. The display of both datasets in real time would have enabled the correlation of abnormal EMG patterns with unstable gait, missteps, or stumbling.

The EMG analysis of the gastrocnemius and soleus muscles in this study did not take into account the complex dynamic interactions between these muscles. The varying forces exerted by the calf muscles during different phases of gait result in interactions and tension within various sections of the Achilles tendon, which are interconnected through the inter-subtendon matrix (ISTM). Recent research has increasingly focused on the structure of the Achilles tendon, with particular attention paid to the semi-independent regions known as Achilles subtendons. Gains et al. (2020) demonstrated that the ISTM allows for independent subtendon movement while ensuring force transmission between the subtendons of the Achilles tendon [45]. To enhance our understanding and quantitatively assess the force transmission between the gastrocnemius and soleus subtendons and their interactions, further research is required.

Lastly, it should be noted that the current findings align with previous research by Handsfield et al. (2016), which emphasized the importance of considering Achilles subtendons as semi-independent regions of the tendon in biomechanical analyses [46].

## 5. Conclusions

The present study demonstrates that even at a speed of 4 km/h with an ankle foot orthosis (AFO), which is within the normal range for walking speeds, there is a greater surface EMG activity of the calf muscles compared to walking at 1 km/h without any orthosis.

The implementation of speed restrictions in clinical practice is a more straightforward process than the prescription of partial weight bearing.

In conclusion, the findings of this study are of particular significance, as they illustrate that modifying walking speed within the normal range has a considerable effect on muscle activity. These insights can be directly applied in clinical practice to optimize patient rehabilitation.

## Figures and Tables

**Table 1:** Experimental protocol.

Condition	Shoe	Angle°/Inserts	Walking Speed (km/h)		
			1	2	4
Full weight	VACOped	0°	√	√	√
Full weight	VACOped	15°	√	√	√
Full weight	VACOped	30°	√	√	√
Full weight	AIRCAST^®^	0 insert	√	√	√
Full weight	AIRCAST^®^	2 inserts	√	√	√
Full weight	AIRCAST^®^	3 inserts	√	√	√
Full weight	Barefoot	0°	√	√	√
Full weight	Sport shoes	0°	√	√	√
Partial load	VACOped	30°	√	√	-
Partial load	AIRCAST^®^	3 inserts	√	√	-
Partial load	Barefoot	0°	√	√	-
Partial load	Sport shoes	0°	√	√	-

**Table 2:** Statistical analysis of the medians of the integral of the gait curve of both orthoses at corresponding angles and velocities for Musculus soleus and Musculus gastrocnemius. A: AIRCAST^®^; V: VACOped.

Condition	Angle 0° Full Load			Angle 15° Full Load			Angle 30° Full Load			Angle 30° Partial Load	
		km/h			km/h			km/h		km/h	
	1	2	4	1	2	4	1	2	4	1	2
M. soleus											
VACOped	1033	991	1170	1119	1196	1140	1175	1188	1571	545	729
AIRCAST^®^	894	1668	1552	853	814	1117	544	630	952	424	465
*p* value	0.171	**0.002**	0.502	0.573	0.184	0.918	**0.001**	**0.020**	0.068	0.434	**0.017**
		**A > V**					**V > A**	**V > A**			**V > A**
M. gastrocnemius											
VACOped	1020	1167	1296	601	808	881	1477	1310	933	254	354
AIRCAST^®^	925	1106	2350	906	1165	1229	826	1083	1487	339	578
*p* value	0.530	0.530	**0.001**	**0.029**	0.110	**0.046**	**0.017**	**0.019**	**0.037**	0.052	**0.024**
			**V > A**	**A > V**		**A > V**	**V > A**	**V > A**	**A > V**		**V > A**

## Data Availability

Data are available upon request from the authors.
